# Association of Severe Thrombocytopenia and Poor Prognosis in Pregnancies with Aplastic Anemia

**DOI:** 10.1371/journal.pone.0103066

**Published:** 2014-07-24

**Authors:** Jae Eun Shin, Young Lee, Sa Jin Kim, Jong Chul Shin

**Affiliations:** Department of Obstetrics and Gynecology, College of Medicine, the Catholic University of Korea, Seoul, Korea; Royal College of Surgeons, Ireland

## Abstract

**Purpose:**

We sought to estimate the risks of adverse obstetric outcomes and disease outcomes associated with severe thrombocytopenia in pregnant women with aplastic anemia (AA).

**Methods:**

In a retrospective study, we compared demographics, clinical characteristics, laboratory results, and outcomes between severe thrombocytopenia (ST) and non-severe thrombocytopenia (non-ST) groups comprising pregnant women with AA.

**Results:**

Of 61 AA patients, 43 (70%) were diagnosed as AA before pregnancy and 18 (30%) were AA during pregnancy. The ST group exhibited lower gestational age at nadir of platelet count (26.0 versus 37.0 weeks, p<0.001) and at delivery (37.3 versus 39.1 weeks, p = 0.008), and a higher rate of bleeding gums (33.8 versus 7.7%, p = 0.015) than the non-ST group. In addition, the ST group exhibited more transfusions during pregnancy (72.7 versus 15.4%, p<0.001) and postpartum period (45.0 versus 2.7%, p<0.001), and more bone marrow transplant after delivery (25.0 versus 0.0%, p<0.001) than the non-ST group. The ST group had a higher odds ratio of composite disease complications (OR, 9.63; 95% CI, 2.82–32.9; p<0.001) and composite obstetric complications (OR, 6.78; 95% CI, 2.11–21.8; p = 0.001) than the non-ST group.

**Conclusions:**

Severe thrombocytopenia is more associated with obstetric and disease complications than is non-severe thrombocytopenia in pregnant women with AA.

## Introduction

Aplastic anemia (AA) is peripheral blood pancytopenia (anemia, neutropenia, and thrombocytopenia) associated with unexplained hypocellularity of the bone marrow. Severe cytopenia may cause life-threatening bleeding and infections. The incidence of AA in Asia is 4–6 per million, which is higher than 2 per million in the Western countries [1]. AA can occur at any age [2], thus young women who were diagnosed with AA during childhood may become pregnant. In addition, pregnancy can be a risk factor for AA through hormonal mechanisms [3]; consequently, pregnant women without any other risk factor can develop AA during the pregnancy. Therefore, information associated with the outcomes of the pregnancy and the disease should be included in patient counseling.

Several case reports of pregnancy associated with AA have been reported [4]–[8]. It has been suggested that pregnancy is associated with AA, in that aplasia may recover spontaneously after abortion or delivery [9]–[11], and preexisting marrow insufficiency may be aggravated during pregnancy [12]–[13]. However, the prognosis and risk factor of complications remain controversial because the rarity of the disease renders it difficult to carry out a detailed analysis in a large-scale study. In fact, some patients experienced spontaneous resolution after delivery, but some died during pregnancy. While some authors have recommended termination in early pregnancy [13], others believe that there is no difference in the prognosis between those undergoing early termination and those continuing the pregnancy [5]. In addition, patients with complete remission had better results than patients with partial remission, but complete remission did not guarantee a good prognosis [4]. The most recent study, which included as many as 36 patients, reported that the risk for a complicated pregnancy in AA after immunosuppression was higher in patients with low platelet counts and paroxysmal nocturnal hemoglobinuria [12].

In this study, the primary objective was to evaluate the relationship between pregnancy and AA by comparing severe thrombocytopenia (ST) and non-severe thrombocytopenia (non-ST) groups in regards to obstetric and disease complications. The secondary objective was to evaluate the overall prognosis of AA in pregnancy.

## Methods

This was a retrospective study of pregnant women diagnosed with AA between January 1996 and December 2009 at Seoul St. Mary’s Hospital and Yeouido St. Mary’s Hospital. A total of 61 pregnant women with aplastic anemia (all Koreans) were recruited. The median age of the patients was 29 years (range, 20 to 35 years) at delivery. Patients were identified through perinatal databases and their medical records were reviewed. Their records were analyzed for demographic information, clinical features, laboratory findings, and obstetric and disease outcomes. We obtained information from the first observed pregnancy. Blood counts were collected before pregnancy, at least once per trimester, and 1, 6, and 12 months after delivery from patients, and from their babies within 3 days after delivery. At our hospital, blood tests were done monthly following diagnosis except in severe cases, in which they were done at weekly intervals. Blood transfusions were performed during pregnancy when necessary to maintain hemoglobin levels of 8 g/dL or higher and platelet counts of 20×10^9^/L or higher. Vaginal delivery was recommended if there was no other indication for cesarean delivery. Induction delivery was usually recommended near term, as platelet transfusion 1–2 hours before delivery is recommended if the platelet counts were below 50×10^9^/L before delivery. For cesarean sections, patients were transfused 1–2 hours before the surgery if their platelet counts were lower than 100×10^9^/L. Disease status was evaluated at 6 months after delivery. Patients were followed through December 2010. This study was approved by the institutional review boards of the Catholic University of Korea (XC10RIMI0010). The institutional review board waived the need for written informed consent from the participants for their information, because this study was retrospective study and data were analyzed anonymously.

All patients of AA before pregnancy and AA during pregnancy were included in this study. AA before pregnancy was defined as when the patient became pregnant after she developed AA, and AA during pregnancy was defined as when the patient was diagnosed as AA during pregnancy. Exclusion criteria were patients with pancytopenia with immunologic etiologies.

AA was defined as the condition involving hypocellular bone marrow and at least 2 of the 3 following laboratory findings: neutrophil count <1.2×10^9^/L, platelet count <70×10^9^/L, and reticulocyte count <60×10^9^/L [14]. Severe thrombocytopenia was defined as a decrease in blood counts to a platelet count <20×10^9^/L during pregnancy, which is one of the criteria for severe AA. Complete remission was defined as hemoglobin concentration >12 g/dL, neutrophil count ≥1.5×10^9^/L, and platelet count ≥150×10^9^/L. Patients who did not meet the hematologic criteria for complete remission but did not require transfusion and who no longer met the definition for severe AA were considered to have partial remission. No response was defined as a hematologic state of severe AA [15].

We described the basic characteristics of pregnant patients with AA. We compared the demographic, clinical, and laboratory data between the ST and non-ST groups. Blood tests during the study period were compared between the ST and non-ST groups. When several measurements were available for a particular period, the lowest value was used. We also compared disease and obstetric complications. The disease complications were transfusion-dependence after delivery, bone marrow transplant (BMT) or immunosuppressive treatment after delivery, and sepsis. Transfusion-dependence after delivery was defined as the state in which the patient required any transfusion 3 months after delivery. The obstetric complications were preeclampsia/eclampsia, preterm delivery, intrauterine growth restriction (IUGR), fetal death, abortion, 5-min Apgar score <7, and neonatal death. Composite disease outcome was defined as the presence of any of the disease complications, and composite obstetric outcome was defined as the presence of any of the obstetric complications.

All statistical analyses were performed using SAS (version 9.1; SPSS Inc., Chicago, Illinois, USA). Patients were compared using Fisher’s exact test for categorical data, and Student’s *t*-test or the Wilcoxon rank sum test for continuous variables. Blood counts during various periods were compared according to ST and non-ST group using the Wilcoxon rank sum test. The results are presented as the means ± standard deviations. To compare the disease and obstetric outcomes between the 2 groups, logistic regression models adjusted for maternal age and postpartum platelet count were used. Adjusted odds ratios (ORs) and 95% confidence intervals (CIs) were calculated.

## Results

In total, 61 pregnant women with AA were included in this study. No patient died during the study period, nor had paroxysmal nocturnal hemoglobinuria. Thirty-four (55.7%) pregnancies were uneventful, which meant no obstetric or disease complications. Obstetric complications were preeclampsia/eclampsia (n = 9, 14.8%), preterm delivery (n = 3, 4.9%), IUGR (n = 3, 4.9%), fetal death (n = 5, 8.2%), abortion (n = 4, 6.6%), 5-min Apgar score <7 (n = 13, 21.3%), and neonatal death (n = 11, 18.0%). Disease complications were long treatment after delivery (n = 14, 23.0%), BMT or chemotherapy after delivery (n = 11, 18.0%), and sepsis (n = 3, 4.9%). Forty-seven (77.0%) patients delivered by the vaginal route and 22 (36.0%) patients became transfusion-dependent during pregnancy. The hematologic conditions of 4 patients (6.6%) were aggravated in the first trimester: 3 aborted, 2 of which were artificial, while one abortion was spontaneous; the remaining patient maintained her pregnancy and successfully delivered in the third trimester. Among 11 patients (18.0%) who were aggravated in the second trimester, 4 delivered in the second trimester and 7 delivered in the third trimester.

Of 61 AA patients, 43 (70%) were diagnosed with AA before pregnancy and 18 (30%) were diagnosed with AA during pregnancy. AA before pregnancy was not statistically different from AA during pregnancy in maternal age of delivery (29 versus 30 years, p = 0.709), gestational age at delivery (39 versus 38.2 weeks, p = 0.152), and ST group (32.7 versus 27.8%, p = 0.713). The postpartum remission state of AA before pregnancy and AA during pregnancy did not differ in that the proportion of no response was not different (4.65% versus 11.1%, p = 0.546). In cases of AA before pregnancy, 30 (70%) women were in complete remission, 10 (23%) were in partial remission, and 3 (7%) were in no response state. All patients with AA during pregnancy had normal blood counts before pregnancy. Among the neonates, 2 received packed red blood cell transfusion because of anemia and recovered thereafter, and 1 patient had transient thrombocytopenia. None of the neonates had pancytopenia.

Table 1 lists the clinical characteristics in the women according ST and non-ST group. The ST group exhibited lower gestational age at nadir of platelet count (26.0 versus 37.0 weeks, p<0.001) and at delivery (37.3 versus 39.1 weeks, p = 0.008), and a higher rate of bleeding gums (33.8 versus 7.7%, p = 0.015) than the non-ST group.

**Table 1:** Clinical characteristics in women with ST group and non-ST group.

	ST group(N = 22)	Non-ST group(N = 39)	*p*-value
Age, *y*	28.5 (23.0–35.0)	30.0 (20.0–35.0)	0.374
Gestational age at nadir of platelet count, *w*	26.0 (6.0–39.0)	37.0 (15.0–40.0)	<0.001
Gestational age at delivery, *w*	37.3 (7.5–40.1)	39.1 (19.1–41.4)	0.008
Cesarean delivery, *n*	7 (31.8%)	7 (17.9%)	0.216
Birth weight, *Kg*	2.9 (0.9–4.3)	3.0 (0.1–4.7)	0.571
Symptom during pregnancy, *n*			
Bleeding gums	7 (33.8%)	3 (7.7%)	0.015
Bruise	12 (54.5%)	21 (53.8%)	0.958
Vaginal bleeding	4 (18.2%)	3 (7.7%)	0.217
Dizziness	2 (9.1%)	1 (2.6%)	0.258
Nasal bleeding	1 (4.5%)	0 (0.0%)	0.179

AA, aplastic anemia; ST, severe thrombocytopenia.

Data are presented as the median (range) or number (percentage) of patients.

Table 2 lists the treatment provided to the women in the ST and non-ST groups. The ST group was associated with more transfusions during pregnancy (72.7 versus 15.4%, p<0.001) and postpartum period (45.0 versus 2.7%, p<0.001), and more BMT after delivery (25.0 versus 0.0%, p<0.001) than the non-ST group.

**Table 2:** Treatment in women with ST group and non-ST group.

	ST group(N = 22)	Non-ST group(N = 39)	*p*-value
Treatment during pregnancy, *n*			
Steroid	2 (9.1%)	1 (2.6%)	0.173
Transfusion	16 (72.7%)	6 (15.4%)	<0.001
Immunsuppressive therapy	1 (4.5%)	0 (0.0%)	0.179
Packed RBC^a^	4.84±6.6	0.40±0.96	<0.001
PC^a^	9.00±19.0	0.19±1.23	0.004
SDP^a^	1.63±4.04	0.02±0.15	0.012
Treatment during labor			
ransfusion	20 (90.9%)	28 (71.8%)	0.080
Packed RBC^a^	3.11±2.78	1.43±1.97	0.010
PC^a^	16.4±28.3	4.67±7.851	0.015
SDP^a^	0.78±1.114	0.55±1.292	0.513
FFP^a^	0.11±0.459	0.00±0.00	0.138
Treatment after delivery			
Steroid	2 (10.0%)	4 (10.8%)	0.924
Transfusion	9 (45.0%)	1 (2.7%)	<0.001
BMT	5 (25.0%)	0 (0.0%)	<0.001
Immunsuppressive therapy	3 (15.0%)	1 (2.7%)	0.083
Packed RBC^a^	0.62±1.586	0.00±0.00	0.016
SDP^a^	0.81±1.47	0.03±0.16	0.002

AA, aplastic anemia; ST, severe thrombocytopenia; CR, complete remission; PR, partial remission, NR, no response, RBC, red blood cell; PC, platelet concentrate; SDP, single donor platelet; FFP, fresh frozen plasma; BMT, bone marrow transplant.

Data are presented as the median (range) or number (percentage) of patients.

a Data are presented as mean ± SD.

**Figure 1:**
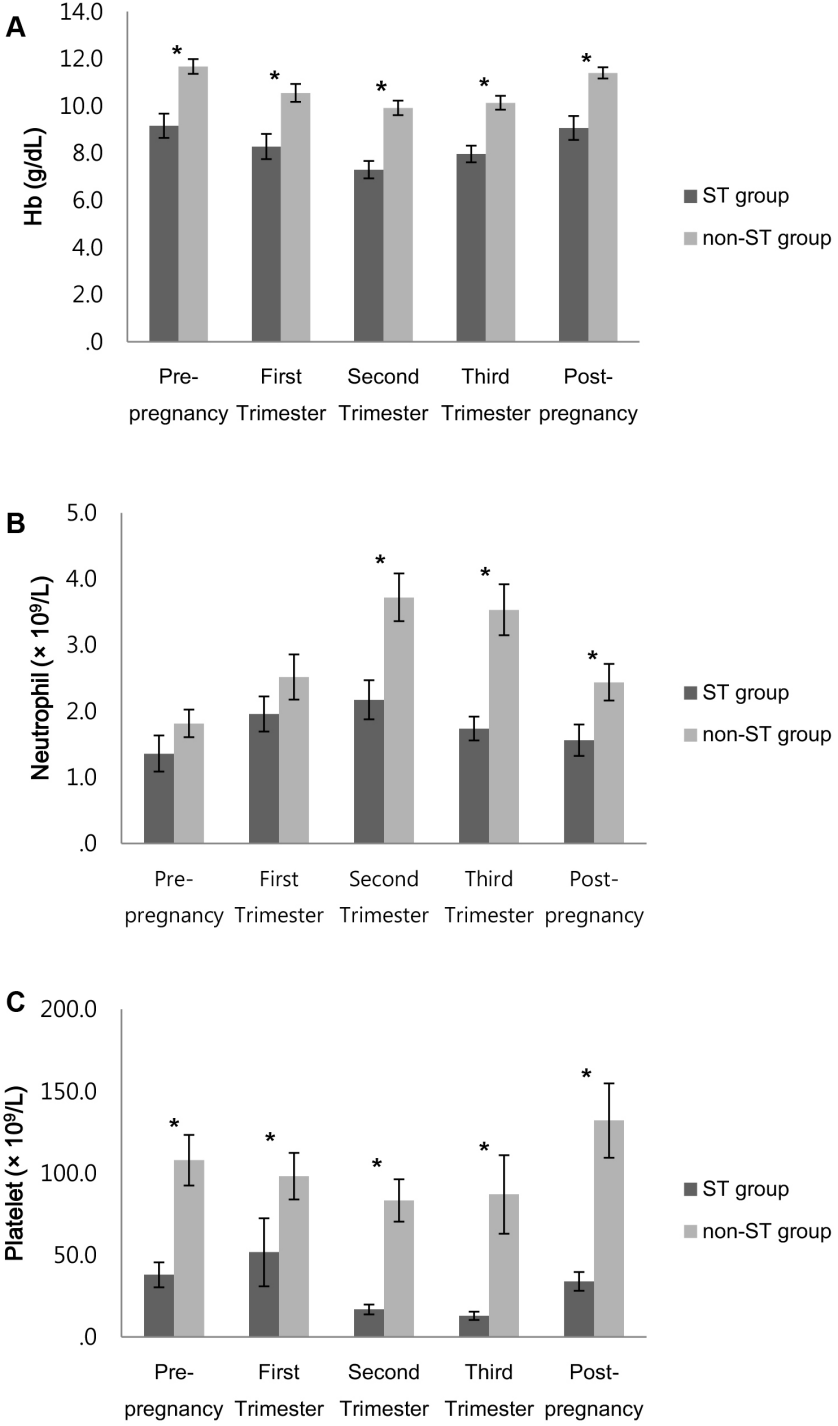
Laboratory findings in women with ST group and non-ST group. (A) Hemoglobin concentration. (B) Neutrophil count. (C) Platelet count. Data are presented as the mean value. Bar represent standard deviation. *p<0.05. Abbreviations: ST, severe thrombocytopenia.

The laboratory findings are compared in Figure 1. The mean hemoglobin levels (g/dL) in the ST and non-ST groups were 9.2 and 11.7 (p = 0.001) in the prepregnancy state, 8.3 and 10.5 (p = 0.001) in the first trimester, 7.3 and 9.9 (p<0.001) in the second trimester, 8.0 and 10.1 (p = 0.001) in the third trimester, and 9.1 and 11.4 (p = 0.002) in the postpartum state, respectively. The mean hemoglobin level of the ST group was lower than that of the non-ST group in all periods, and hemoglobin levels decreased during pregnancy and normalized to the prepregnancy state at the postpartum state.

The mean neutrophil count (×10^9^/L) in the ST and non-ST groups were 1.4 and 1.8 (p = 0.144) in prepregnancy state, 2.0 and 2.5 (p = 0.171) in the first trimester, 2.2 and 3.7 (p = 0.014) in the second trimester, 1.7 and 3.5 (p = 0.0003) in the third trimester, and 1.6 and 2.4 (p = 0.023) in the postpartum state, respectively. In the 2 groups, the mean neutrophil count was statistically different in the second and third trimesters, and the postpartum state, and neutrophil counts increased during pregnancy and normalized to the prepregnancy state at the postpartum state.

The platelet count (×10^9^/L) in the ST and non-ST groups were 38.0 and 107.9 (p = 0.003) in the prepregnancy state, 51.8 and 98.2 (p = 0.002) in the first trimester, 16.9 and 83.4 (p<0.0001) in the second trimester, 13.0 and 87.0 (p<0.0001) in the third trimester, and 34.0 and 132.1 (p<0.0001) in the postpartum state, respectively. The mean platelet counts of the ST group were lower in all of the states, and platelet counts decreased during pregnancy and normalized at the postpartum state.

There were no significant differences in reticulocyte count in both groups (1.72% in ST group versus 52.9% in non-ST group, P = 0.329).

The composite disease and obstetric complications were compared between the 2 groups (Table 3). The ST group had a higher odds ratio of composite disease complications (OR, 9.63; 95% CI, 2.82–32.9; p<0.001) and composite obstetric complications (OR, 6.78; 95% CI, 2.11–21.8; p = 0.001) than the non-ST group. After controlling for age and postpartum platelet count, the ST group maintained a higher odds ratio of composite disease complications (OR, 11.0; 95% CI 2.67–45.3; p = 0.001) and composite obstetric complications (OR, 5.75; 95% CI, 1.50–22.1; p = 0.011). Regarding the disease complications, AA pregnant women with severe thrombocytopenia faced higher odds of transfusion-dependence after delivery (OR, 54.9; 95% CI, 6.33–475; p = 0.000) and BMT or immunosuppressive treatment after delivery (OR, 5.24; 95% CI, 1.44–19.0; p = 0.012). Regarding the obstetric complications, AA pregnant women with severe thrombocytopenia faced higher odds of preeclampsia/eclampsia (OR, 21.7; 95% CI, 2.49–190; p = 0.005).

**Table 3:** Composite disease and obstetric outcomes.

		Unadjusted OR			Adjusted OR^a^		
		OR	(95% CI)	*p*-value	OR	(95% CI)	*p*-value
bComposite disease outcome	Non-ST groupST group	1.00			1.00		
		9.63	2.82–32.9	<0.0001	11.0	2.67–45.3	0.001
cComposite obstetric outcome	Non-ST groupST group	1.00			1.00		
		6.78	2.11–21.8	0.001	5.75	1.50–22.1	0.011

Values are expressed as odds ratio (95% confidence interval).

a Adjusted by age and postpartum platelet count.

Abbreviations: OR, odds ratio; CI, confidence interval.

b Defined as any of the following: transfusion-dependence after delivery, bone marrow transplant or immunosuppressive treatment after delivery, or sepsis,

c Defined as any of the following: preeclampsia/eclampsia, preterm delivery, intrauterine growth restriction (IUGR), fetal death, abortion, 5-min Apgar score <7, or neonatal death.

## Discussion

Our data in pregnant women with AA suggested that severe thrombocytopenia was more associated with obstetric and disease complications than non-severe thrombocytopenia.

Traditionally, severe AA is diagnosed when the patient has hypocellular bone marrow and at least 2 of 3 laboratory abnormalities, which comprise reticulocyte, neutrophil, and platelet counts. Previous studies have applied the same prognostic factors of severity for pregnant women. Deka argued that maternal and fetal outcome is poor in severe AA [16]. On the contrary, Suda reported no hematologic differences between successful and unsuccessful groups [8]. Those results are conflicting not only because they involved small sample sizes, but also because they applied the same prognostic factor as overall AA without special consideration of pregnancy.

In overall AA, the severity of neutropenia may affect prognosis, as infectious disease is known to be the major cause of death in severe AA [17]. Although hemorrhage and sepsis are believed to be the main reasons for death and morbidity in pregnant women with AA, sepsis occurred rarely in the present study. Moreover, blood tests revealed a comparative increase in neutrophil counts during pregnancy, unlike the platelet and hemoglobin counts, which decreased during pregnancy. It may explain why patients rarely developed sepsis and their neutrophil counts did not decrease to the level of neutropenia, which would have required prophylactic administration of granulate colony–stimulating factors. Another study also reported that neutrophil counts increased during pregnancy and that thrombocytopenia was one of the risk factors for AA in pregnancy [12]. Our study identified severe thrombocytopenia as a risk factor for pregnant patients with AA. Therefore, we believe platelet counts should serve as the main risk factor in evaluations of the severity of AA in pregnant women for AA before as well as during pregnancy.

In cases of severe AA, some authors have recommended termination [13], as continuation of such a pregnancy may jeopardize maternal health. Our study proved that successful pregnancies were possible in women with severe thrombocytopenia. One of 3 and 7 of 11 women who were diagnosed as having severe thrombocytopenia in the first and second trimester, respectively, maintained their pregnancies and delivered at term with intensive supportive care. A previous study also reported that more than 50% of pregnancies were uneventful after treatment of AA with immunosuppression in AA before pregnancy, which was similar to our result [12]. One-third of the patients became transfusion-dependent during pregnancy, and 19% experienced relapse of AA, but most patients with relapse recovered spontaneously or after repeated immunosuppressive therapy. Regarding AA during pregnancy, pregnancy was possible without complications, except transfusions [18]. Therefore, continuation of pregnancy, rather than early termination, is recommended. However, numerous transfusions may be required; therefore, patients should be informed of this risk, as a large number of transfusions may run the risk of BMT failure due to the risk of transfusion refractoriness [19].

It has been suggested that the outcome of pregnancy and maternal survival were better in women who had AA before pregnancy as compared to those in whom it developed during pregnancy [8], [20]. However, our study demonstrated that there are no differences between the 2 groups with regards to severity and postpartum condition. Patients with severe AA might not conceive because they are already aware of the risk of relapse during pregnancy, and patients with AA before pregnancy can prepare for the risks and undergo regular check-ups. Conversely, patients with AA during pregnancy did not have risk factors before conception, thus the prognosis might be poor. Nevertheless, they were not different statistically with regards to the possibility of severe criteria during the pregnancy and remission state 6 months after delivery.

Due to the bleeding tendency in thrombocytopenia, some authors have recommended cesarean delivery because of the possibility for spontaneous intracranial hemorrhage due to labor. Tichelli demonstrated that cesarean sections were more frequent in women who experienced a relapse of AA or symptomatic thrombocytopenia [12]. Others have recommended vaginal delivery because it involves less blood loss than cesarean delivery [16], [21]. In our hospital, no patient experienced intracranial hemorrhage even while struggling during labor. Induction delivery permits timely transfusions before delivery for safety and less bleeding during vaginal delivery, and preoperative transfusions before cesarean sections could decrease complications as well.

There are some limitations to our study. First, as a retrospective study, certain data were irretrievable because of missing records. Moreover, as a retrospective study, there could have been inherent selection bias. Second, prognosis was better than that in previous studies possibly because a patient not in the remission state might not attempt to conceive in view of previous reports about the risk of maternal death and morbidity.

Despite such limitations, this study included the largest number of patients with AA treated in 2 hospitals of the same university, which, to our knowledge, follow the same protocol. In addition, we demonstrated the current prognosis and treatment protocol of pregnant women with AA that reflect the advances of medicine in supportive treatment. This research is meaningful in that this data can be helpful for counseling women who are contemplating pregnancy in AA before pregnancy and those who happen to be diagnosed for the first time during pregnancy.

Our study showed that pregnancy can be successful in a patient with AA, but continuation of the pregnancy should be individualized in that the risk factors of complication should be considered with the informed consent of the patients and their families.
